# Extracorporeal membrane oxygenation therapy for pulmonary decompression illness

**DOI:** 10.1186/cc13935

**Published:** 2014-06-20

**Authors:** Yutaka Kondo, Masataka Fukami, Ichiro Kukita

**Affiliations:** 1 Department of Emergency Medicine, Graduate School of Medicine, University of the Ryukyus, 207 Uehara, Nishihara, Okinawa 903-0215, Japan

## 

A 36-year-old man presented with tetraplegia and hyperventilation after diving. On arrival, he was in shock and an echocardiogram showed air bubbles in the right ventricle. Pulmonary decompression illness was suspected and hyperbaric oxygen therapy (100% oxygen, 2.8 ATA for 60 minutes) was administered. However, the patient’s respiratory status gradually worsened. He also showed right ventricle load D-shape during cardiac ultrasonography, which was attributed to the presence of air bubbles. Chest radiograph revealed a bilateral butterfly shadow (Figure 1). Because the patient’s oxygen saturation level was almost 60%, we decided to initiate extracorporeal membrane oxygenation (ECMO) therapy [1]. Considering that the patient had a hyperdynamic cardiac function and that bubbles generally diminish within 24 hours, we selected venovenous ECMO therapy rather than venoarterial ECMO therapy. This therapy was surprisingly effective, and the patient’s respiratory failure gradually ameliorated. Venovenous ECMO was discontinued on day 7, when a chest X-ray revealed only a small area of consolidation.

**Figure 1:**
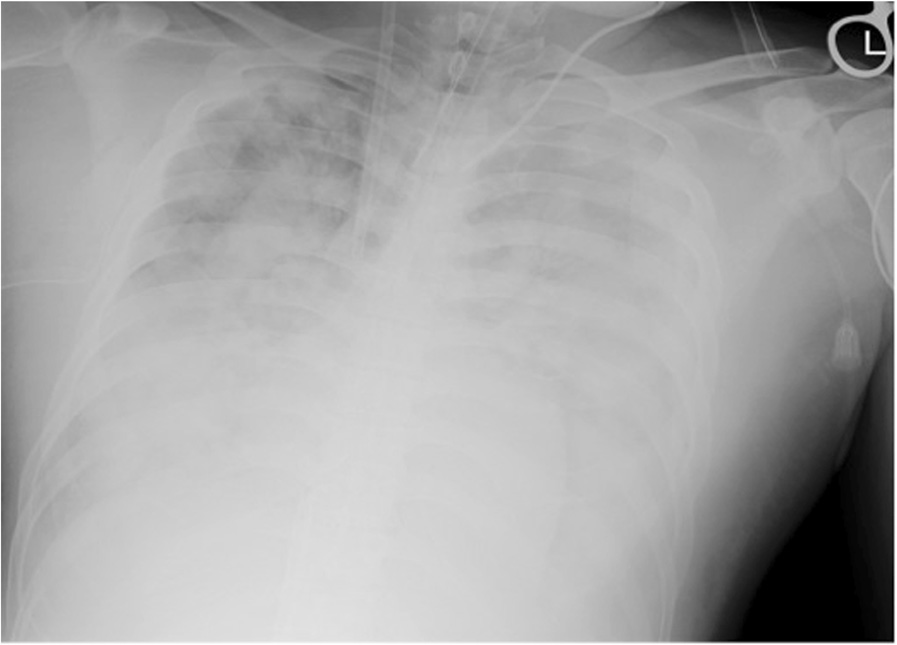
Chest radiograph at the time of initiating extracorporeal membrane oxygenation therapy.

Pulmonary decompression illness is rarely observed in clinical settings, and most patients die prior to hospitalization [2,3]. We administered ECMO therapy to rescue the patient, even though this therapy has rarely been reported with good outcome in patients with decompression illness. In addition, we had to select venovenous ECMO even with the patient showing right ventricular failure. A lot of physicians may select venoarterial ECMO if the patient shows right ventricular failure, but the important physiological mechanism of pulmonary decompression illness is massive air embolism in the pulmonary arteries, and the bubbles diminish within the first 24 hours. The management of decompression illness therefore differs substantially from the usual right-sided heart failure.

ECMO may be considered as one of the treatments for severe decompression illness.

## Abbreviations

ATA: Atmospheres absolute; ECMO: Extracorporeal membrane oxygenation.

## Competing interests

The authors declare that they have no competing interests.
